# Enhancing prime editing via inhibition of mismatch repair pathway

**DOI:** 10.1186/s43556-022-00072-5

**Published:** 2022-02-23

**Authors:** Lurong Jiang, Shaohua Yao

**Affiliations:** Laboratory of Biotherapy, National Key Laboratory of Biotherapy, Cancer Center, West China Hospital, Sichuan University, Renmin Nanlu 17, Chengdu, 610041 Sichuan China

## Background

In the recent issue of Cell [1], Chen et al. from David Liu laboratory discovered that inhibition of DNA mismatch repair (MMR) significantly enhanced the efficiency of prime editing while abated the frequency of unintended indels. Prime editing (PE) is currently the most precise and versatile genome editing technique that allow all kinds of base conversions and small fragment deletion or insertion in the genome [2], but its efficiency is generally lower than that of other Cas9 derived editing tools, such as nuclease Cas9 and base editing [3].

Prime editing was also invented in David Liu laboratory by fusing MMLV- reverse transcriptase (RT) to Cas9 nickase. Noteworthy, since the Nobel-winning work demonstrated that Cas9 system is reprogrammable for new target [4], it was soon widely used in the biomedical research because of its superiority over former reprogrammable nucleases. Basically, the Cas9 system consists two effectors, the Cas9 protein and its guide RNAs, crRNA (CRISPR RNA) and tracrRNA (trans-activating CRISPR RNA), which can be engineered into one single guide RNA (sgRNA). Under the guidance of sgRNA, wildtype Cas9 protein recognizes its target DNAs and produces double strand breaks. Mutant Cas9 protein with impaired nuclease activity can still bind the targets, which can be used as a platform to recruit other effectors to perform localized DNA manipulations. Cas9 disassociates the non-target strand (NTS) of sgRNA from its target strand (TS) to form a R-loop structure and the disassociated NTS is exposed outside complex and therefore can serve as a substrate for enzymes preferring ssDNAs. This feature was well utilized in designing base editors [3], where a cytosine or adenine deaminase was fused with Cas9 nickase that specifically cleaves the TS. By deaminating the cytosines or adenines in the exposed NTS, these editors converted them into uracil and hypoxanthine respectively, which were then converted into thymine and guanine respectively with the assistance of endogenous repair mechanisms. Although effective, the accuracy and versatility of base editors are limited in that they non-selectively converted their substrate bases within the editing window and they mainly perform base transitions.

**Fig. 1:**
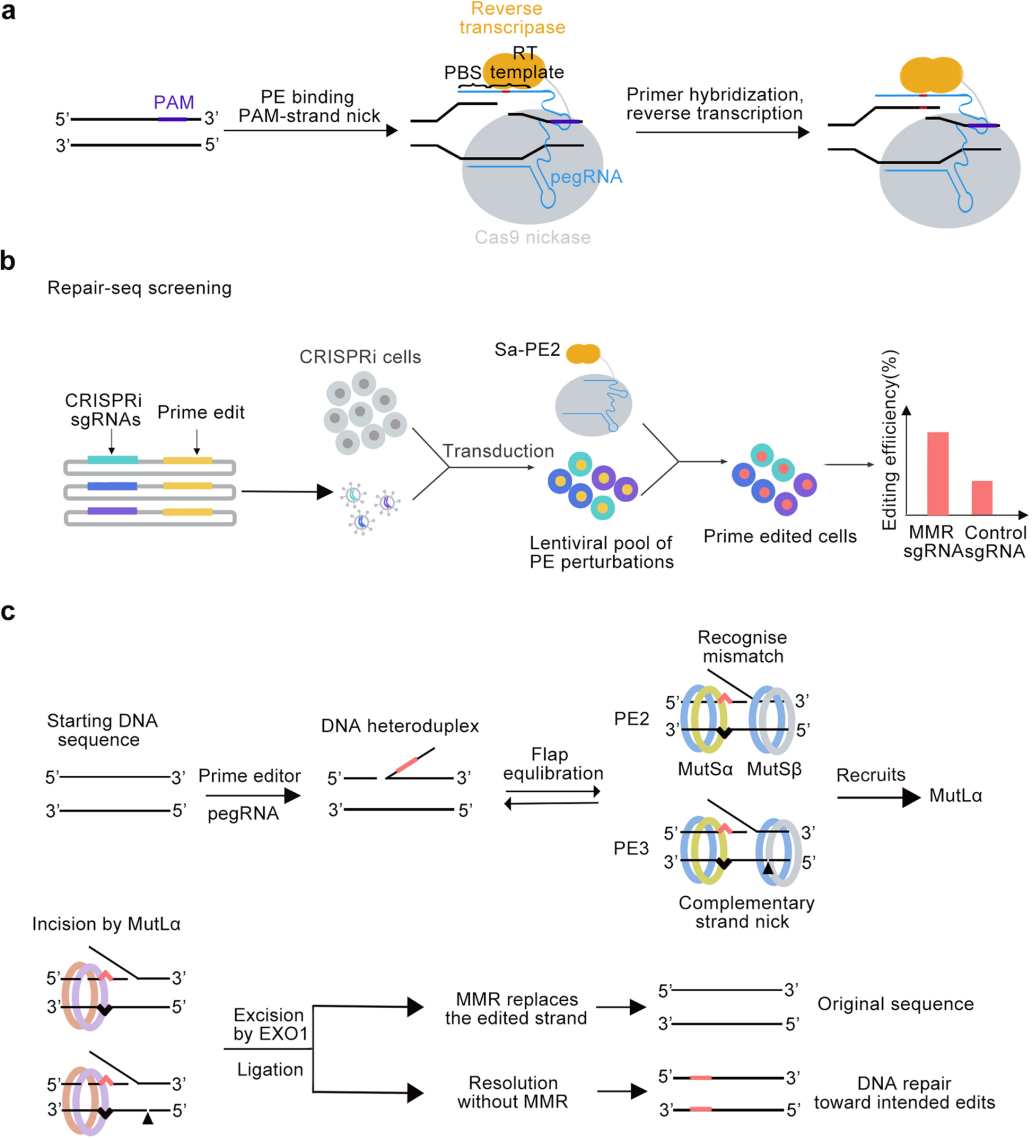
**a** Overview of prime editing. **b** The patterns of MMR Repair-seq screening. **c** Model for DNA mismatch repair (MMR) of PE intermediates. This figure is completely original.

To resolve these issues, David Liu laboratory developed prime editors, which utilize Cas9 nickase to cleave NTS, prime editing sgRNA (pegRNA) to bind the TS, and RT to reverse transcribe the 3’ end of NTS according to the information encoded by pegRNA. These concerted actions ultimately produced a 3’ flap with intended edits, inducing endogenous DNA repair mechanisms to integrate the edits into the genome. Armed with well-designed pegRNAs, PE enables virtually all types of editing, including point mutations, deletions and insertions. However, the efficiency of PE is generally lower than that of other Cas9 based tools (Fig. 1a).

In the recent Cell paper, David Liu laboratory discovered a practical solution to enhance the efficiency of PE The authors conducted a pooled CRISPRi screening with sgRNAs targeting genes involved in DNA repair and identified that interfering MMR genes enhanced both the efficiency and accuracy of prime editing. In their screening system (Fig. 1b), named Repair-seq, *Streptococcus pyogenes Cas9* (SpCas9) derived transcriptional repressor (dCas9–KRAB) was used to couple with pooled suppressor sgRNAs to inhibit gene expression and *Staphylococcus aureus* Cas9 (SaCas9) derived prime editor (Sa-PE2) was used to prime edit the target sequence that was inserted downstream of each suppressor sgRNA. Therefore, the effect of each suppressor sgRNA on the editing of target is readily determined by deep sequencing. The screening revealed that sgRNAs targeting key players in DNA mismatch repair (MMR) pathway, including MSH2, MSH6, MLH1, and PMS2, increased the prime editing efficiency of SaPE2. This finding was further supported by siRNA knockdown and endogenous loci. A model was proposed to explain the effect (Fig. 1c), in which MMR recognized the intermediate heteroduplex that was formed by the pairing of PE-extended 3’ flap and TS. Because the 3’ flap was accompanied with a nick, it tended to be excised out of the edited strand by MMR and the original, unedited TS was kept intact. Interestingly, the level of enhancement by MMR inhibition was dependent on cell types, which might due to varied MMR activities in these cells, indicating that the effect of MMR inhibition on PE depends on the state of MMR of the target cells. The level of enhancement seemed also correlated with the types of PE, because it declined as the length of the indel loops increased, which might possibly due to the limitations of MMR in recognizing unpaired regions (1-13nt). However, considering the fact that one of the two DNA lesion sensors of MMR, MutSβ complex, specifically binds at the double-strand/single-strand junction of branched substrates, including 3’ flap structure, to facilitate their cleavage, it would be reasonable to deduce that inhibition of MMR would protect the PE-extended 3’flap at its up-paired state, thereby generally benefiting for all types of PEs. The authors went further by incorporating a genetic encoded MLH1 inhibitor, dominant negative mutant MLH1dn (truncated MLH1 D754–756 lacking endonuclease domain), to prime editor to form PE4 (PE2 + MLH1dn) or PE5 system (PE3 + MLH1dn), which showed enhanced editing efficiency and reduced indels across a variety of cell types. Importantly, no obvious microsatellite instability was observed, suggesting that transient action of PE4 or PE5 is relative safe. However, this observation did not rule out the possibility of increasing general mutation rate, which deserves carefully examinations.

In summary, Chen et al. have demonstrated that inhibition of MMR enhanced the efficiency of PE. The authors provided systematic investigation and detailed evidence to demonstrate the effects of MMR pathway on the outcomes of prime editing. Based on these findings, they also developed genetic encoded and high-efficient prime editors, PE4 and PE5, which shall enhance the potential of this technology for clinical translation.

## Data Availability

Not applicable.
